# Effects of a Range-Expanding Sea Urchin on Behaviour of Commercially Fished Abalone

**DOI:** 10.1371/journal.pone.0073477

**Published:** 2013-09-20

**Authors:** Elisabeth M. A. Strain, Craig R. Johnson, Russell J. Thomson

**Affiliations:** 1 Scienze Ambientali, Università di Bologna, Ravenna, Italy; 2 Institute for Marine and Antarctic Studies, University of Tasmania, Hobart, Tasmania, Australia; University of Shiga Prefecture, Japan

## Abstract

**Background:**

Global climate change has resulted in a southerly range expansion of the habitat modifying sea urchin *Centrostephanus rodgersii* to the east coast of Tasmania, Australia. Various studies have suggested that this urchin outcompetes black-lipped abalone (*Haliotis rubra*) for resources, but experiments elucidating the mechanisms are lacking.

**Methodology/Principal Findings:**

We outline a new framework involving experimental manipulations and Markov chain and Pareto modelling to examine the effects of interspecific competition between urchins and abalone and the effect of intraspecific competition in abalone, assessed as effects on behaviour. Manipulations of abalone densities had no detectable effect on urchin behavioural transitions, movement patterns or resightability through time. In contrast, additions of urchins resulted in abalone shifting microhabitats from exposed to sheltered positions, an increase in the proportion of mobile abalone, and declines in abalone resightability through time relative to controls without the urchins. Our results support the hypothesis of asymmetrical competitive interactions between urchins and abalone.

**Conclusions/Significance:**

The introduction of urchins to intact algal beds causes abalone to flee and seek shelter in cryptic microhabitat which will negatively impact both their accessibility to such microhabitats, and productivity of the abalone fishery, and will potentially affect their growth and survival, while the presence of the abalone has no detectable effect on the urchin. Our approach involving field-based experiments and modelling could be used to test the effects of other invasive species on native species behaviour.

## Introduction

Global climate change is resulting in the poleward range expansion of many marine species [1], [2], [3], [4]. Range expanding species can have negative impacts on native species by altering biotic interactions, habitat complexity, environmental chemistry and other physical variables [5], [6], [7]. The paucity of information about the nature and effects of interactions between many range expanding and native species limits understanding of potential impacts of range expansions on marine ecosystems, and hinders efforts to prioritise management responses [8].

The southeast coast of Australia has experienced a marked increase in the abundances of some warmer water species [9], [10] as a result of greater poleward penetration of the East Australia Current [11]. The long-spined sea urchin (*Centrostephanus rodgersii*) has extended its range from New South Wales to Tasmania [9], [12], [13], [14]. The establishment of the species is of particular concern because of its ability to catastrophically overgraze productive and diverse algal beds, and maintain an alternative and stable bare rock barrens habitat [15], resulting in local declines in the abundances of commercially fished black-lipped abalone (*Haliotis rubra*) [13].

The long-spined sea urchin and black-lipped abalone are the largest macro-invertebrate herbivores on shallow rocky reef habitat in southeast Australia, and they share similar habitat, predators and dietary preferences [13], [16], [17]. Both species predominately remain in cryptic habitat during the day, moving out into the open at night to feed on drift and attached filamentous and foliose algae [16]. Surveys along the south east coast of Australia have demonstrated a negative relationship between the abundances of the urchin and black-lipped abalone at a broad range of spatial scales [13], [17]. The urchins appear to have a negative effect on abundances of abalone through exploitative competition for food and by overgrazing of canopy algae and forming barrens habitat [18], [19]. However this research was conducted in experimental enclosures and in barrens habitat which could have amplified or altered competitive interactions between urchins and abalone by limiting their access to drift algae resources or preferred habitat [18], [19].

While the impacts of long-spined urchins on abalone in barrens habitat are relatively clear [19], here we outline a new framework which involves experimental manipulations of urchin and abalone densities within open (i.e. unfenced) experimental plots in intact algal beds. The approach was designed to test the effects of interspecific competition on urchins and the effects of intra- and interspecific competition on abalone behaviour, defined as discrete changes in microhabitat occupied [20], [21]. We used Markov chain modelling [20] to test the effects of competition on abalone and urchin behavioural transitions over time, a method that has been used extensively in epidemiology and econometrics, but less so in behavioural ecology [21]. Log linear regression and a Pareto distribution (used to test for evidence of Levy flight) [22] were used to determine the effects of competition on abalone and urchin movement patterns. Our aims were to test (1) whether removal of abalone from open plots results in long-spined urchins spending more time exposed, moving less distance, and greater local abundance within plots relative to control plots containing abalone, and (2) whether additions of urchins and/or extra abalone to open plots results in abalone spending more time sheltered or outside the plot, increases in distances moved and declines in the abundances of abalone in treatment plots relative to control plots without urchins and extra abalone.

## Methods

### Ethics statement

No ethics permits were required for the described study, which complied with all relevant regulations. Permission to work in the Maria Island marine reserve and at the Lanterns site was granted by the Department of Primary Industry, Parks, Water and Environment Tasmania. The study did not use any endangered or protected species.

### Site characteristics

Experiments were conducted on subtidal reefs at two sites, Magistrates Point (148.02°E, 42.58°S) and the Lanterns (147.57°E, 42.52°S), ∼60 km apart on the east coast of Tasmania, between June 2006 to March 2007. Both sites support diverse seaweed stands of several canopy-forming algal species (dominated by *Ecklonia radiata*) with a well-developed understorey, but at the Lanterns there are also small patches of urchin barrens created and maintained by the long-spined sea urchin (*Centrostephanus rodgersii*).

We tested the nature and effects of interactions between urchins and abalone at Magistrates Point in the Maria Island marine reserve to avoid confounding with any effects from fishing of abalone, fish and rock lobsters. The reserve supports high densities of abalone (at the time of the experiments, 25.0±SE  = 0.95 individuals per 9 m^2^, with shell length ranging between 90–90 mm) but very low densities of long-spined urchins (0.5±SE  = 0.11 individuals per 9 m^2^, with test diameter ranging between 85–23 mm) relative to the surrounding fished areas [23]. All urchins used in the experiments at Magistrates Point were collected from the Lanterns which supports a relatively high density of long-spined urchins (18.0±SE  = 0.98 individuals per 9 m^2^, with test diameter ranging between 90–25 mm).

The site at Magistrates Point is characterised by a gently sloping rocky substratum to a depth of ∼11 m and is moderately sheltered from all but south-westerly swells. All experiments were conducted in the depth range 7–11 m where preliminary surveys at the Lanterns demonstrated the distribution of urchins and abalone overlapped.

### Experimental plots

Our experimental manipulations were conducted in 3×3 m unfenced plots. An area of 9 m^2^ is well within the average distances moved by urchins and abalone over weekly periods [24], [25]. All plots were marked with 5 star pickets, 1 in each corner and 1 in the centre of the plot. In each plot, we assessed the cover of different types of substrata, algae and sessile invertebrates by eye, and counted the number of macroinvertebrate grazers (Table S1).

### Tagging urchins and abalone

In all experimental treatments we tagged urchins and abalone with unique identifiers excepting those animals in the tagging control treatments (see Experiments 3 and 4). Abalone were tagged *in situ* by scrubbing their shell with a copper wire brush to remove macroscopic fouling organisms and then gluing leader sheep ear tags (12×2 mm) to the shell with epoxy resin, which was mixed immediately prior to diving (Z-spar A-788). Urchins were collected at the Lanterns and tagged immediately on the dive vessel, using previously developed methods which have no detectable impacts on urchin behaviour, growth or survival [18]. Urchins translocated to Magistrates Point from the Lanterns were transported in large bins containing seawater by car and boat over ∼3 hours before being transferred randomly into the plots. For the animals that remained at the Lanterns, tagged urchins were handled in a similar manner before being returned to the water and released into plots.

### Experimental manipulations

We conducted four experiments (*n* = 3 replicates of each treatment) to test the effects of interspecific competition and the translocation procedures on urchins and the effects of intra- and interspecific competition and the tagging procedure on abalone (see Table 1 for details). Experiments 1, 3 and 4 were conducted in winter 2006 and Experiment 2 in summer 2007 to test for any seasonal differences in the effects of adding urchins on abalone. For all the experiments, 1× ambient densities of urchins was 18 individuals per 9 m^2^ (average densities at the Lanterns) and 1× ambient densities of abalone was 25 individuals per 9 m^2^ (average densities at Magistrates Point) and thus 2× ambient densities of abalone was 50 individuals per 9 m^2^ (maximum densities at Magistrates Point).

**Table 1:** Summary details of the experiments, planned comparisons and results to test the effect of interspecific competition and translocation procedures on the urchins, and the effect of intra- and interspecific competition and the tagging procedure on abalone behaviour, movement and percentage resighted through time.

Experiment	Comparison	Treatment	Time Points	Dependent variables measured	Results
1	18U0A vs. 18U25A	Removal of abalone	Treatment and control: 1 day; 1, 2 and 4 weeks	Urchins distance moved; percentage resighted; and behaviour transitions	No detectable effects
	0U25A vs. 18U25A	Additions of urchins	Treatment and control: 1 day; 1, 2 and 4 weeks	Abalone distance moved; percentage resighted; and behaviour transitions	Increase in sheltering behaviour, distances moved, and decrease in percentage resighted
2	0U25A vs. 0U50A	Addition of extra abalone	Control at weeks 1, 2, 3 and 7, 8, 9. Treatment at weeks 4, 5, 6	Abalone distance moved; percentage resighted; and behaviour transitions	Increase in sheltering and distances moved
	0U25A vs. 18U25A	Addition of urchins	Control at weeks 1, 2, 3 and 7, 8, 9. Treatment at weeks 4, 5, 6	Abalone distance moved; percentage resighted; and behaviour transitions	Increase in sheltering behaviour, distances moved, and decrease in percentage resighted
3	T1 vs. T2	Tagging	Control and treatment at 1 day; 1, 2 and 4 weeks	Urchins percentage resighted	No detectable effects
	T2 vs. T3	Translocation within plot	Control and treatment at 1 day; 1, 2 and 4 weeks	Urchins percentage resighted	No detectable effects
	T3 vs. T4	Translocation to a new plot at the same site	Control and treatment at 1 day; 1, 2 and 4 weeks	Urchins percentage resighted	No detectable effects
	T4 vs. T5	Translocation to a new site	Control and treatment at 1 day; 1, 2 and 4 weeks	Urchins percentage resighted	No detectable effects
4	1A^no_tag^ vs. 1A	Tagging	Control and treatment at 1 day; 1, 2 and 4 weeks	Abalone percentage resighted	No detectable effects

0U, 18U: 0× ambient density of urchins and 1× ambient density of urchins and 0A, 25A, 50A: 0× ambient density of abalone, 1× ambient density of abalone and 2× ambient density of abalone. Treatment codes are: T1 = 1U untagged, unmanipulated in plots at the Lanterns, T2 = 1U tagged, placed back into the same positions and plots at the Lanterns, T3 = 1U tagged, random positions, same plots at the Lanterns, T4 = 1U tagged, placed randomly into new plots at the Lanterns, T5 = 1U tagged, placed randomly into new plots at Magistrates Point. 1A^no tag^  = A untagged in plots at Magistrates Point.

#### Experiment 1

To test the effects of interspecific competition on urchins and abalone the following treatments were applied to plots at Magistrates Point:

Treatment 1: 1× ambient density of urchins were added to randomly selected positions in plots from which all abalone had been removed (18U0A).

Treatment 2: 1× ambient density of urchins were added to randomly selected positions in plots with 1× ambient density of abalone (18U25A).

Treatment 3: 1× ambient density of abalone with no urchins (0U25A).

#### Experiment 2

To test the effects of intra- and interspecific competition on abalone the following treatments were applied to plots at Magistrates Point.

Treatment 1: 1× ambient density of tagged abalone with no urchins (0U25A).

Treatment 2: 1× ambient density of tagged abalone added to 1× ambient density of tagged abalone, with no urchins (0U50A).

Treatment 3: 1× ambient density of tagged abalone with 1× ambient density urchins (18U25A).

The additional abalone for treatment 2 were collected approximately 100 m away from the experimental plots, tagged by marking the shell with orange crayon (which lasted the duration of the experiment) and placed randomly into plots. We did not monitor the responses of the extra abalone in treatment 2. This experiment ran for a total of 9 weeks; weeks 1 to 3 were monitored prior to addition of urchins or extra abalone to provide a baseline, for weeks 4 to 6 we added urchins or extra abalone and them removed the added animals at the end of the period, and weeks 7 to 9 were after the removal of the urchins and extra abalone in week 3. This experiment was run as a ‘before *versus* after’ comparison to assess the effects of adding and removing both sea urchins and extra abalone on the responses of resident abalone.

#### Experiment 3

To test the effect of interspecific competition on urchins, it was necessary to translocate tagged urchins from the Lanterns to Magistrates Point, and thus it was necessary to control for both the tagging and translocation procedures [26]. In winter of 2006, the following treatments were applied to plots at the site where the urchins were collected at the Lanterns, and at Magistrates Point.

Treatment 1: 1× ambient density of untagged and unmanipulated urchins left in plots at the Lanterns.

Treatment 2: 1× ambient density of tagged urchins removed from and then, after tagging and retaining in large bins containing seawater for ∼3 hours, placed back into the same positions and plots from which they were collected at the Lanterns.

Treatment 3: As for treatment #2 but 1× ambient density of tagged urchins were placed in random positions into the same plots from which they were collected at the Lanterns.

Treatment 4: Similar to Treatment #3, but 1× ambient density of tagged urchins were placed randomly into new plots at the Lanterns.

Treatment 5: 1× ambient density of tagged urchins were placed randomly into new plots at Magistrates Point (transportation time in large bins with seawater ∼3 hours).

#### Experiment 4

To test the effects of the tagging procedure on abalone the following treatments were applied to plots at Magistrates Point:

Treatment 1: 1× ambient density of tagged abalone (25A).

Treatment 2: 1× ambient density of untagged abalone (25A^no_tag^).

### Response variables

For all experiments, the number and identity of urchin and abalone predators, including southern rock lobster (*Jasus edwardii*) and demersal fishes (*Pictilabrus laticlavius, Notolabrus tetricus, Notolabrus fucicola* and *Latridopsis forsteri*), inside and to 1 m outside each plot were recorded by divers within the first 5 minutes. We then recorded the number of tagged urchins and abalone (including any dead animals) inside and to 1 m outside each plot and the number of untagged abalone inside the plot.

In Experiments 1 and 2 the behaviour and movement of urchins and abalone within each plot were recorded. Behaviour was described as either exposed (located out in the open) or sheltered (within a crevice, under a rock, or sitting vertically against a rock, usually in some kind of ‘corner’). Animals within a 1 m zone outside the plots were recorded as being outside, and tagged animals that were not relocated at a particular time (because they were deep in the crevices of the reef matrix, or outside of the search area and could not be observed without moving the boulder substratum) were classified as lost. The position of individual urchins and abalone inside plots was determined by triangulation based on the distances to the 2 nearest star pickets out of the five star pickets in each plot, and noting which picket was on the right hand side when facing the plot. Movement of urchins and abalone was described by their change in position on consecutive visits to plots.

In Experiments 1, 3 and 4 our assessments of urchins and abalone behaviour, movement and local abundances, and the identity and abundances of predators took place immediately prior to, and 1 day, 1, 2 and 4 weeks after, the initial manipulations. In Experiment 2, our assessments of urchins and abalone behaviour and local abundances, and the identity and abundances of predators took place immediately prior to manipulations and then weekly. However, due to time constraints movement of urchins and abalone were only measured during weeks 3, 4, 5, 6.

Experiments 1, 3 and 4 ran for 4 weeks while Experiment 2 ran for a total of 9 weeks; weeks 1 to 3 were preceding the manipulation (prior to the addition of urchins and abalone), weeks 4 to 6 were during treatment (with added urchins or extra abalone), and weeks 7 to 9 were after the cessation of the treatment (i.e. after the urchins and abalone that had been added to the plots were removed). Experiment 1 was run for 4 weeks and was designed to test the effects of interspecific competition on abalone and urchin behaviour, and Experiment 2 was run for 9 weeks to test the effects and recovery from inter- and intraspecific competition on abalone behaviour. Divers spent one hour at each plot on each occasion. All experimental sampling times for recording urchin and abalone behaviour were chosen arbitrarily as we had no prior information about the effects of competition on their behaviour.

### Data analysis

#### Percentage resighted

The effects of interspecific competition and the translocation procedures on the percentage of urchins resighted through time, and the effects of intra- and interspecific competition and the tagging procedures on the percentage of abalone resighted through time, were analysed using 2-way univariate repeated measures ANOVA. We used the Greenhouse-Geisser adjusted degrees of freedom when data did not meet the assumption of sphericity. To test the effects of interspecific competition on urchins, the model included the main effects of treatment (fixed, 2 levels  = 18U0A, 18U25A), time (random, 4 levels  =  day 1, weeks 1, 2, 4) and their interaction. Similarly, to test the effect of interspecific competition on abalone, the model included the main effects of treatment (fixed 2 levels  = 0U25A, 18U25A), time (random, 4 levels  =  day 1, weeks 1, 2, 4) and their interaction. To test the effects of intra- and interspecific competition on abalone, the model included the main effects of treatment (fixed, 3 levels  = 0U25A, 0U50A, 18U25A), time (random, 9 levels  =  weeks 1 to 9) and their interaction. To test the effect of the translocation procedures on the percentage of urchins resighted through time, the model included the main effects of treatment (fixed, 5 levels  = 5 treatments, for details see Experiment 3), time (random, 4 levels  =  day 1, weeks 1, 2, 4) and their interaction. Similarly, the effect of the tagging procedures on the percentage of tagged abalone resighted through time, the model included the main effect of treatment (fixed, 2 levels  = 2 treatments, for details see Experiment 4), time (random, 4 levels  =  day 1, weeks 1, 2, 4) and their interaction.

#### Behavioural transitions

The effect of interspecific competition on urchins and intra- and interspecific competition on abalone behavioural transitions (the probability of changing from one behavioural state to another) were analysed using Markov chain modelling. Markov chains quantify the dependence of a given behaviour on preceding behaviour. There are several degrees of dependence; if sequenced behaviours are independent they are described by a zero-order Markov chain, while if a particular behaviour depends only on the behaviour immediately preceding it, then a first-order Markov chain is fitted, and so on. Here we used a first-order Markov chain to model abalone and urchin behaviour through time, to ensure that the model is not over-fitted while retaining a relatively simple analytical design. We tested between the zero- and first-order chains for the control animals using a *χ* ^2^ likelihood ratio test.

To create the first order Markov chain model, we used a log linear regression model with a multinomial error distribution. The model included the main effects of urchin and abalone behaviour in the immediately preceding time point, treatment, plot and their interactions. To test the assumption that the behaviour of animals in the control treatment was consistent over the observation period, we included time. A *χ* ^2^ likelihood ratio test was used to test between models with and without time included.

Transition probabilities describing changes in behaviour through time were determined for both urchins and abalone from the regression coefficients of the log-linear model, by solving for *p_ij_* in the following sixteen equations.
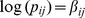


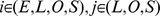


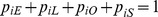


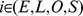
where *i* is the preceding behaviour and *j* the succeeding behaviour (*i* and *j* could include any of the 4 behavioural states; E, L, O or S); *β_ij_* is the coefficient for the *i*th behaviour of the outcome variable and *j*th behaviour of the dependent variable; and *p_ij_* is the probability of being at state *j* at time *t+1*, given the state was *i* at time *t*, which is the transition probability from *i* to *j* in the Markov chain. The effect of interspecific competition on urchins and intra- and interspecific competition on abalone behaviour-transition probability matrices were tested using a *χ* ^2^ likelihood ratio test. A proportions test was used to test for the effect of treatments on specific behavioural transitions.

#### Distance moved

The effects of competition on urchin and abalone movement patterns was analysed in two ways, using a repeated measures log-linear method to examine the effects of competition on total distances moved by urchins and abalone, and using a truncated Pareto distribution to examine the effects of competition on the shape of the distribution of the distances by urchins and abalone. To obtain a measure of the error inherent in the methods used for determining distances moved by urchins and abalone, we measured the distances between the eight pairs of star pickets ten times. The distances between the eight pairs of reference markers were calculated using triangulation and the variability between measurements averaged (0.1 m ± SE  = 0.012 m). Thus there was high precision in our measurements between the reference markers.

To test the effects of competition on urchins and abalone we labelled animals that moved ≤0.4 m per week as sedentary, while those that moved >0.4 m per week were deemed to be mobile. This decision was based on histograms of movement (Figure S1) which showed that the majority of urchins and abalone moved ≤0.4 m (65.8% urchins and 77.5% abalone). We also trialled other arbitrary definitions of sedentary (e.g. ≤0.2 m and ≤0.6 m), but this had no effect on our conclusions. We tested the effect of treatment on the average distances moved by urchins and abalone using a non-parametric method, which showed similar trends (data not shown).

To test the effects of interspecific competition on urchin and abalone movement (day 1, weeks 1, 2, 4) and intra- and interspecific competition on abalone movement (weeks 4, 5, 6), we examined the proportion of sedentary and mobile individuals using a repeated measures log-linear method (using Generalised Estimating Equations). No assumptions were made about the correlation between time points (unstructured correlation structure). The model included the main effects of treatment and plot, and Wald's Test was used to test the significance of these effects. Wald's statistic (W) was calculated by dividing the treatment co-efficient by its standard error and significance assessing by comparing these results to the normal distribution. For the overall test of plot, *W* was based on the linear combination of the coefficients for each plot. Where significant differences were found, planned comparisons were made at specific time points using G-tests (see Table 1 details). To compensate for increased type I error rates and low absolute counts, Williams's corrections (*q*) were applied in all G-tests.

We modelled the distances moved by urchins and abalone, using Pareto, exponential, truncated, and truncated exponential distributions. Truncation was based on the minimum and maximum detectable movement of 0.1 m and 4 m, respectively. Using model selection techniques (AIC and BIC), the truncated Pareto distribution was the best fit model for all movement data. A maximum likelihood method was used to estimate the shape parameter of the distribution for all treatments and at each time point. α^2^ likelihood ratio tests were used to test the effects of interspecific competition on urchins and abalone, and effects of intra- and interspecific competition on abalone on the shape of these distributions.

#### Predators

The densities of urchins and abalone predators were consistently low through time. Therefore, we averaged the densities of rock lobster and demersal fishes through time, for each plot. The differences in the densities of predators between treatments were analysed using 1-way ANOVAs. The models included the main fixed effect of treatment (see above details).

For all parametric ANOVAs, the relationship between the standard deviations and means of the densities from treatment groups was used to determine the appropriate transformation to stabilise variances. Transformed data were checked for both normality (using normal probability plots) and homoscedasticity. Transformations are expressed in terms of the raw dependent variable, *Y*.

For all analyses where significant differences were found in the overall ANOVA, planned comparisons among selected treatments were conducted (Table 1). For Experiment 2, to limit the number of comparisons we pooled the data into weeks 1–3 (preceding the addition of urchins or extra abalone), weeks 4–6 (during treatment with added urchins or extra abalone) and weeks 7–9 (succeeding treatment after urchins or extra abalone were removed). Where comparison sets were non-orthogonal, we adjusted the significance levels [27].

All statistical analyses and graphics were undertaken using the R statistical package (http://www.R-project.org).

## Results

### Percentage resighted

In total, the responses of 108 urchins (*Centrostephanus rodgersii*) and 693 abalone (*Haliotis rubra*) were monitored throughout the experiments. There were no detectable effects of the translocation procedures or of interspecific competition on the percentage of urchins resighted through time (f_1, 8.328_ = 1.238, p>0.05). There were also no detectable effects of the tagging procedure on the percentage of abalone resighted through time (Table 2).

**Table 2:** Results of 2-way repeated measures ANOVA testing the effect of intra- and interspecific competition and the tagging procedure on the percentage of tagged abalone resighted in experiments at Magistrates Point, Maria Island.

Experiment	Factors	df	MS	F	P	Comparisons	F	P
1.	Treatment	1	1539.4	18.501	**0.013**	0U25A vs.18U25A day 1	2.99	0.159
	Error	4	83.206			0U25A vs.18U25A week 1	40.913	**0.003**
	Time	2.082	304.872	5.163	**0.034**	0U25A vs.18U25A week 2	15.814	**0.01**
	Treatment × Time	2.082	179.839	3.045	0.101	0U25A vs.18U25A week 4	10.61	**0.01**
	Error	8.328	59.055					
2.	Treatment	2	1386.289	3.01	0.124	0U25A weeks 1–3 vs. weeks 4–6	4.192	0.06
	Error	6	460.569			0U25A weeks 1–3 vs. weeks 7–9	3.828	0.07
	Time	2.958	1509.364	15.38	**<0.001**	1U1A weeks 1–3 vs. weeks 4–6	16.941	**0.001**
	Treatment × Time	5.915	398.702	4.063	**0.01**	1U1A weeks 1–3 vs. weeks 7–9	22.299	**<0.001**
	Error	17.746	98.141			0U2A weeks 1–3 vs. weeks 4–6	10.868	0.01
						0U2A weeks 1–3 vs. weeks 7–9	7.819	0.02
						1U1A weeks 4–6 vs. 0U2A weeks 4–6	43.898	**<0.001**
4.	Treatment	1	45.594	0.762	0.432			
	Error	4	59.865					
	Time	1.89	221.668	3.291	0.0.95			
	Treatment × Time	1.89	66.365	0.985	0.412			
	Error	7.598	67.349					

Significant p-values are shown in bold: p<0.05 are significant for the main analysis and p<0.0125 are significant for the planned comparisons testing the effect of interspecific competition and p<0.007 are significant for the planned comparisons testing the effect of intra- and interspecific competition (α adjusted using Todd and Keough 1994). See Table 1 for the full explanation of treatment codes.

**Figure 1:**
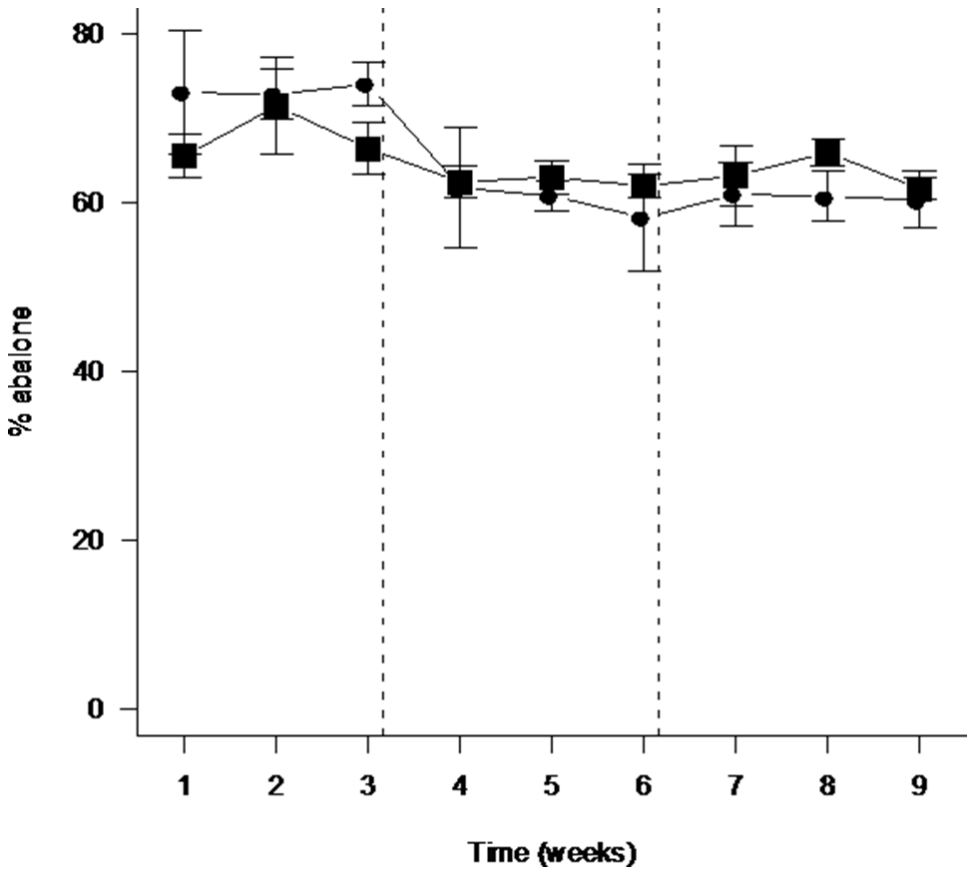
Effect of interspecific competition on the percentage of tagged abalone resighted through time (weeks), in Experiment 2, at Magistrates Point, Maria Island. Data are means (+/−SE) of *n* = 3 replicate plots. Squares are 0U18A  = 1× ambient density abalone (weeks 1–9) and circles are 18U25A  = 1× ambient density abalone with 1× ambient density urchins (weeks 1–3 were prior to adding urchins, weeks 4–6 with added urchins and weeks 7–9 after the urchins were removed). There were significant differences between 0U25A (weeks 1–3) vs. 18U25A (weeks 4–6), 18U1A (weeks 1–3) vs. 18U25A (weeks 7–9), (see Table 2).

In contrast, interspecific competition resulted in significant declines in the percentage of abalone resighted through time (Figure S2, Figure. 1, Table 2). For the interspecific competition experiment, additions of urchins resulted in significant declines in the percentage of abalone resighted in weeks 1, 2 and 4 when compared with control plots without urchins (Figure S2, Table 2). However there were no detectable effects of interspecific competition on the percentage of abalone resighted only 1 day after the urchins were added (Figure S2, Table 2). For the intra- and interspecific competition experiment, there were no detectable changes in the percentage of abalone resighted through time in the control treatment or in the treatment with added abalone (Figure 1, Table 2). However, additions of urchins resulted in a decline in the percentage of abalone resighted in weeks 4 to 6 (with urchins), and in weeks 7 to 9 (following the removal of the urchins), relative to the initial period (weeks 1 to 3) prior to the addition of the urchins (Figure 1, Table 2). Inter- rather than intraspecific competition explained the significant decline in the percentage of abalone resighted through time after addition of the urchins to plots (Figure 1, Table 2).

### Behavioural transitions

**Figure 2:**
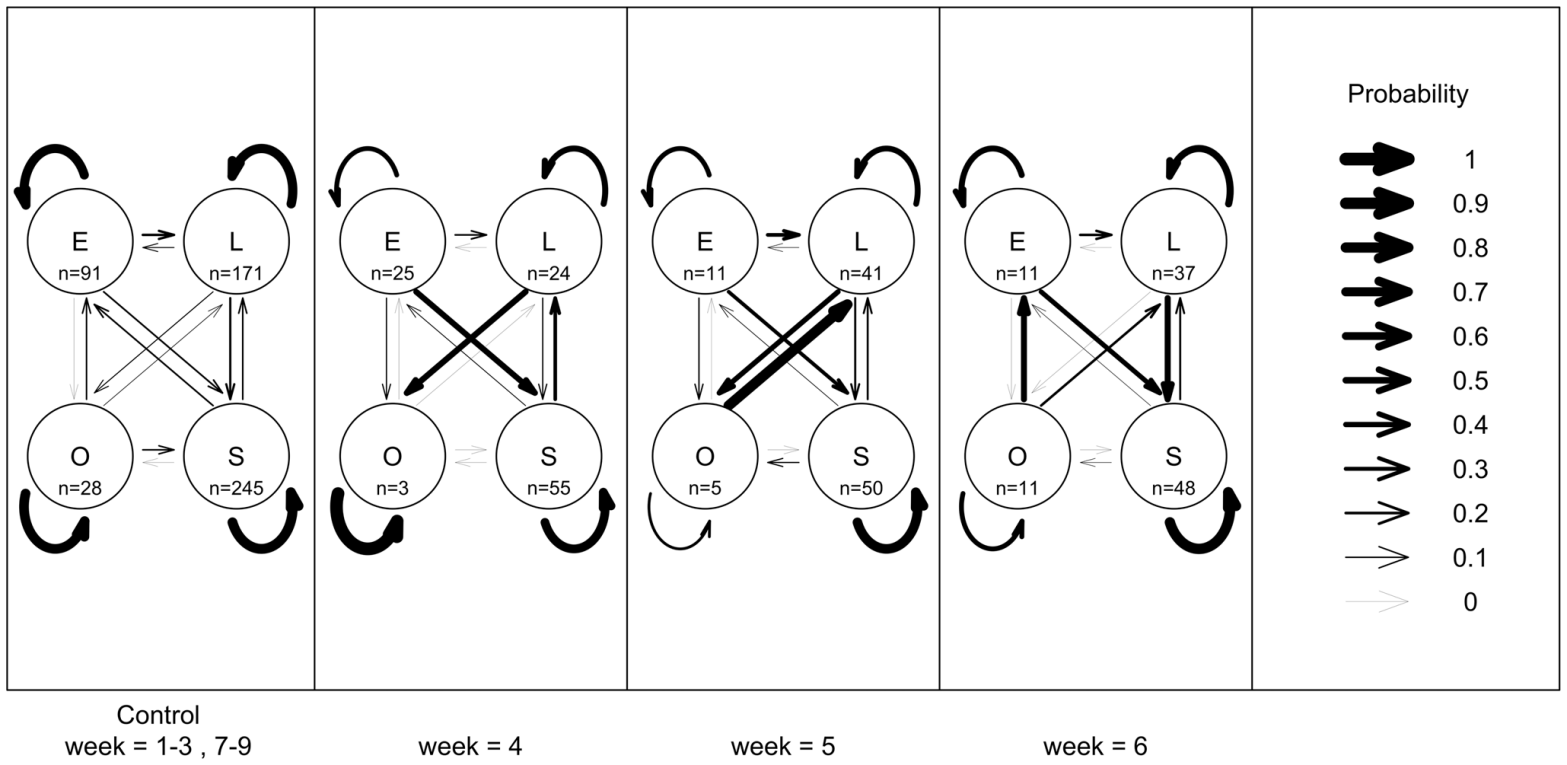
Effects of interspecific competition on abalone behavioural transitions, in Experiment 2 at Magistrates Point, Maria Island. Data are the proportion of tagged abalone observed in each behavioural state the week before (E =  exposed, L =  lost, O =  outside the plot and S =  sheltered) in 3 replicate plots. (a) 0U25A: 1× ambient density abalone, (weeks 1–3 prior to adding urchins and weeks 7–9 after urchins were removed), (b) 18U25A: 1× ambient density urchins, with 1× ambient density abalone, (week 4 with added urchins), (c) 18U25A: 1× ambient density urchins with 1× ambient density abalone, (week 5 with added urchins), and (d) 18U25A: 1× ambient density urchins ×1 ambient density abalone, (week 6 with added urchins). The numbers of abalone in a given behavioural state are summed across all times for the control and given separately at each time for the treatment and are represented as “n = .” inside each circle. Arrows of different thickness are used to show the relative probabilities of abalone transitioning from each behavioural state. The straight arrows show the probabilities of abalone transitioning from one behavioural state to another (e.g. E to S) and the curved arrows show the probabilities of abalone ‘transitioning’ to the same behavioural state (e.g. E to E). These proportions sum to 1 for a given behaviour state (e.g. E-E, E-S, E-L, E-O).

**Figure 3:**
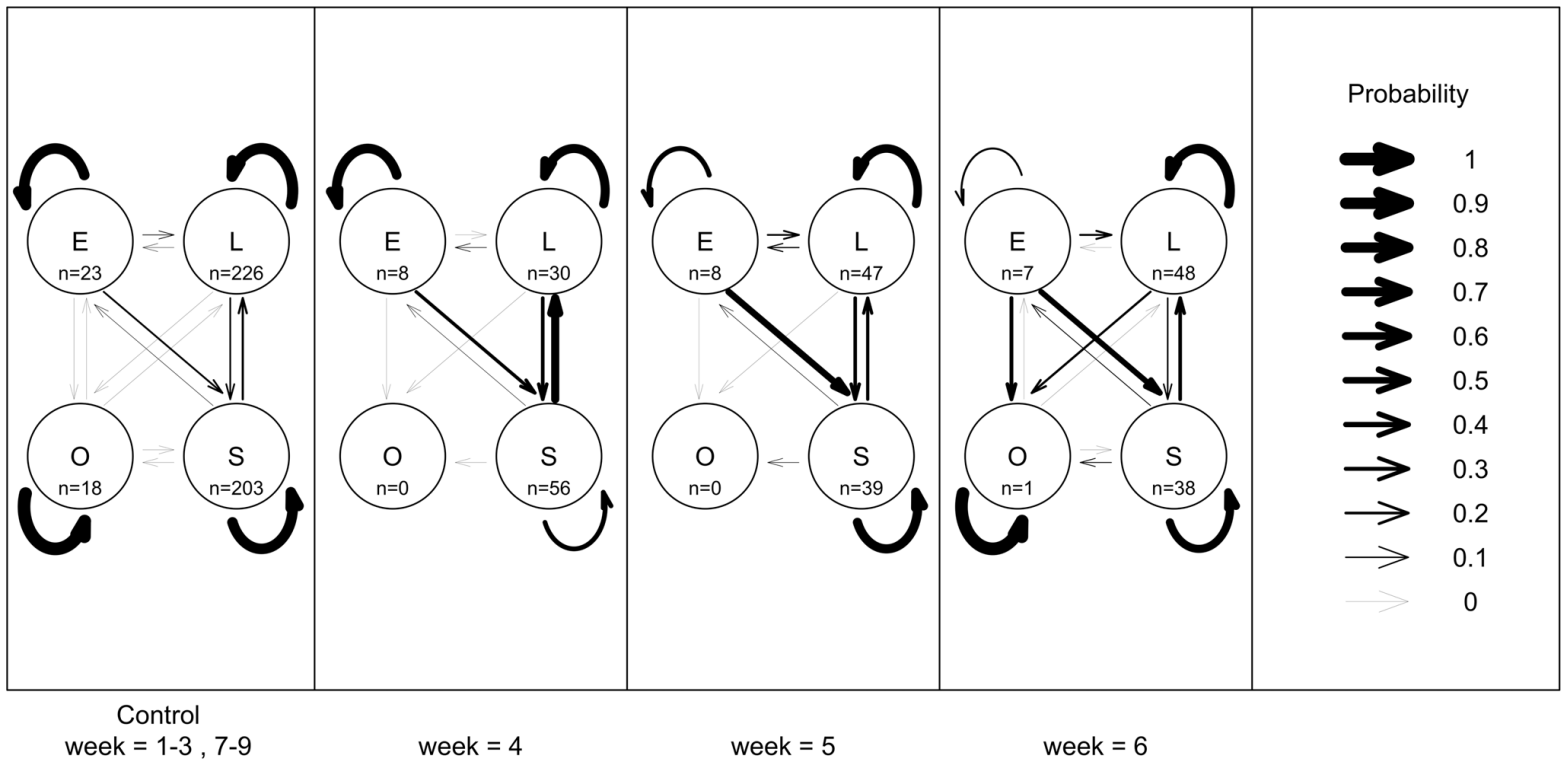
Effect of intraspecific competition on abalone behavioural transitions, in Experiment 2, at Magistrates Point, Maria Island. Data are the proportion of tagged abalone observed in each behavioural state the week before (E =  exposed, L =  lost, O =  outside the plot and S =  sheltered) in *n* = 3 replicate plots. (a) 0U18A: 1× ambient density abalone, (weeks 1–3 prior to adding extra abalone and weeks 7–9 after the extra abalone were removed combined), (b) 0U50A: 2× ambient density of abalone (week 4 with added extra abalone), (c) 0U50A: 2× ambient density of abalone, (week 5 with added extra abalone), and (d) 0U50A: 2× ambient density of abalone (week 6 with added extra abalone). The numbers of abalone in a given behavioural state are summed across all times for the control and given separately at each time for the treatment and are represented as “n = .” inside each circle. Arrows of different thickness are used to show the relative probabilities of abalone transitioning from each behavioural state. The straight arrows show the probabilities of abalone transitioning from one behavioural state to another (e.g. E to S) and the curved arrows show the probabilities of abalone ‘transitioning’ to the same behavioural (e.g. E to E). These proportions sum to 1 for a given behaviour state (e.g. E-E, E-S, E-L, E-O).

Transitions among the behavioural states of urchins (α^2^_24_  = 13.1, p = 0.97) and abalone (interspecific competition experiment: α^2^_33_  = 43, p = 0.11; intra- and interspecific competition experiment: α^2^_5_  = 0.78, p = 0.85) in the control treatment were stable through time. Therefore, for the control plots the transitions among the behavioural states of urchins and abalone were combined across all time points (Figure S3, Figures. 2, 3). The first-order Markov chain model provided more information than the zero-order model for the behaviour states of urchins and abalone in the controls (interspecific competition experiment: Urchins, α^2^_9_  = 108.5, p<0.001, Abalone, α^2^_9_  = 534.4, p<0.001, and intra- and interspecific competition experiment: α^2^_60_  = 378.7, p<0.001). There were no detectable differences between the plots for the controls (interspecific competition experiment: Urchins, α^2^_24_  = 20.6, p = >0.05, Abalone, α^2^_24_  = 34.7, p>0.05 and intra- and interspecific competition experiment: Abalone, α^2^_75_  = 81.4, p>0.05).

There were no detectable effects of interspecific competition on urchin behavioural transitions (α^2^_15_  = 8.71, p>0.05). In contrast, additions of urchins resulted in immediate and significant changes to abalone behavioural transitions (interspecific competition experiment: α^2^_12_  = 21.3, p<0.001, Figure S3, and intra- and interspecific competition experiment: α^2^_3_  = 3.857, p = 0.03) (Figure. 2), while additions of extra abalone resulted in significant but slower and less consistent changes to abalone behaviour (α^2^_3_ = 14.138, p<0.001) (Figure. 3). One week after the addition of urchins to plots, the probability of abalone remaining exposed decreased significantly relative to the control without urchins (interspecific competition experiment: p = 0.003, Figure S4, intra- and interspecific competition experiment: p = 0.002, Figure 2) and remained consistently low while the urchins were present. The probability of abalone changing their behaviour from exposed to sheltered significantly increased a week after the addition of urchins, (interspecific competition experiment: p = 0.001, intra- and interspecific competition experiment: p = 0.039). Similarly the probability of abalone remaining sheltered significantly increased a week after the addition of urchins (interspecific competition experiment: p = 0.041, intra- and interspecific competition experiment: p = 0.03). The probability of abalone shifting from sheltered to exposed significantly declined within one week after the addition of urchins (interspecific competition experiment: p = 0.039, intra- and interspecific competition experiment: p = 0.004) and remained consistently low throughout the 1 month experiment relative to the control (Figure S3, Figure 2).

One week after extra abalone were added to plots, the probability of abalone remaining sheltered significantly decreased (intra- and interspecific competition experiment: p = 0.001) relatively to control plots without extra abalone. Three weeks after the addition of extra abalone the probability of abalone remaining in shelter increased (intra- and interspecific competition experiment: p = 0.04) (Figure 3). Two weeks after extra abalone were added to plots, the probability of abalone remaining exposed significantly decreased (intra- and interspecific competition experiment: p = 0.017) and remained at this low level while the extra abalone were present relative to the control (Figure 3).

### Distance moved

**Figure 4:**
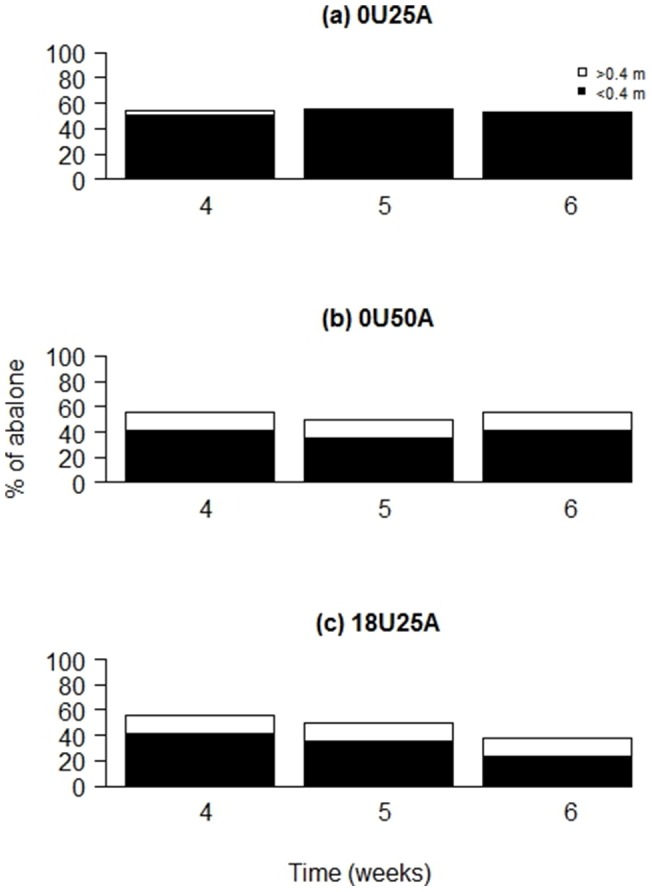
Effect of intra- and interspecific competition on the percentage of sedentary (≤0.4 m) and mobile (>0.4 m) abalone through time (weeks) in Experiment 2 at Magistrates Point, Maria Island. (a) 0U25A: 1× ambient density of abalone with no urchins, (b) 18U25A: 1× ambient density of urchins with 1× ambient density of abalone, and (c) 0U50A: 2× ambient density of abalone with no urchins. Black bars are sedentary abalone (distance moved ≤0.4 m) and white bars are mobile abalone (net distances moved >0.4 m). There were significant differences between 0U18A vs. 0U50A in weeks 5 and 6 and 0U25A vs. 18U25A in weeks 4, 5 and 6 (see Table 3 results).

In both experiments, there was no detectable effect of plot on the movement patterns of abalone (interspecific competition experiment: W = −0.24, p = >0.05; intra- and interspecific competition experiment: W = −0.39, p>0.05). Thus, we excluded plot from the model. There were no detectable effects of competition on distances moved by urchins (interspecific competition experiment: W = −0.28, p>0.05). In contrast, additions of urchins and extra abalone significantly affected the proportion of mobile abalone (interspecific competition experiment: W = 2.84, p = 0.004; intra- and interspecific competition experiment: W = 4.65, p<0.001). In the interspecific competition experiment, additions of urchins resulted in an increase in the proportion of mobile abalone in weeks 1, 2 and 4, compared with control plots without urchins (Figure S4, Table 3). For the intra- and interspecific competition experiment, additions of urchins (weeks 4–6) resulted in an increase in the proportion of mobile abalone compared with control plots without urchins (Figure. 4, Table 3). Additions of extra abalone also led to increases in the proportion of mobile abalone in weeks 5 and 6, when compared with control plots without extra abalone (Figure 4, Table 3). However, there were no detectable effects of extra abalone on the proportion of mobile abalone in week 4 relative to control plots (Figure 4, Table 3). There was no detectable difference in the effect of intra- and intraspecific competition on the proportion of mobile and sedentary abalone in weeks 4, 5 and 6 (Figure 4, Table 3).

**Table 3:** Results of G-tests testing the effect of interspecific and intra- and interspecific competition on the proportion of sedentary (≤0.4 m per week) and mobile (>0.4 m per week) abalone in experiments at Magistrates Point, Maria Island.

Experiment	Comparisons	Time	g-test statistic	P
1.	0U25A vs.18U25A	Day 1	2.314	0.128
	0U25A vs.18U25A	Week 1	5.746	**0.01**
	0U25A vs.18U25A	Week 2	6.541	**0.01**
	0U25A vs.18U25A	Week 4	6.917	**0.008**
2.	0U25A vs. 18U25A	Week 4	10.31	**0.002**
	0U25A vs. 18U25A	Week 5	8.846	**0.002**
	0U25A vs. 18U25A	Week 6	15.685	**<0.001**
2.	0U25A vs. 0U50A	Week 4	5.79	0.01
	0U25A vs. 0U50A	Week 5	10.134	**0.001**
	0U25A vs. 0U50A	Week 6	16.964	**<0.001**
2.	18U25A vs. 0U50A	Week 4	0.136	0.713
	18U25A vs. 0U50A	Week 5	0.07	0.8
	18U25A vs. 0U50A	Week 6	0.734	0.39

Significant p-values are shown in bold print: p<0.0125 are significant comparisons testing the effect of interspecific competition and p<0.005 are significant comparisons testing the effect of intra- and interspecific competition (α adjusted using Todd and Keough 1994). See Table 1 for the full explanation of treatment codes.

All movement data were fitted to the truncated Pareto distribution (Table 4). In both Experiment 1 addressing interspecific competition and Experiment 2 addressing intra- and interspecific competition simultaneously, there was a highly significant effect of plot on the movement patterns of urchins and abalone (interspecific competition at day 1, and intra- and interspecific competition at weeks 4 and 6) (Table 5). While the results were consistent through the whole experiment, we tested the effects of interspecific competition on the movement patterns of urchins and abalone at week 1 and the effects of interspecific competition on the movement patterns of abalone were tested at week 5, to avoid any confounding effects of plot.

**Table 4:** Parameter estimation, after fitting a Pareto distribution to the movement steps of abalone.

Experiment	Treatment	Time	Mean distance moved	N	Parameter estimate (µ_t_)
1.	0U25A	Day 1	0.304	59	5.37
	0U25A	Week 1	0.33	53	4.73
	0U25A	Week 2	0.311	53	4.98
	0U25A	Week 4	0.261	51	6.46
1.	18U25A	Day 1	0.41	61	3.15
	18U25A	Week 1	0.506	52	2.87
	18U25A	Week 2	0.575	48	2.63
	18U25A	Week 4	0.621	45	2.3
2.	0U25A	Week 4	0.226	54	15.5
	0U25A	Week 5	0.216	57	25.8
	0U25A	Week 6	0.202	54	99.6
2.	18U25A	Week 4	0.471	56	3.02
	18U25A	Week 5	0.537	51	2.89
	18U25A	Week 6	0.497	55	3.06
2.	0U50A	Week 4	0.554	41	2.92
	0U50A	Week 5	0.461	30	3.42
	0U50A	Week 6	0.512	22	2.57

See Table 1 for the full explanation of treatment codes.

**Table 5:** Results of likelihood ratio test of the effects of interspecific and intra- and interspecific competition on the distribution of abalone movement fitted with a Pareto distribution in the experiments at Magistrate's Point, Maria Island.

Experiment	Test	Time	Likelihood Ratio	P
1.	0U25A vs. 18U25A	Day 1	1414	**<0.001**
	0U25A vs. 18U25A	Week 1	388	**<0.001**
	Plot Effect	Day 1	155	0.039
	Plot Effect	Week 1	3.1	0.69
	Plot Effect	Week 2	6.84×1024	**<0.001**
	Plot Effect	Week 4	1.65×109	**<0.001**
2.	0U25A vs. 18U25A	Week 4	2.42×10^12^	**<0.001**
	0U25A vs. 0U50A	Week 4	3.26×10^9^	**<0.001**
	0U50A vs. 18U25A	Week 4	1.21	0.53
	Plot Effect	Week 4	30.3	0.34
	Plot Effect	Week 5	5.36×10^7^	**<0.001**
	Plot Effect	Week 6	7.55×10^5^	**<0.001**

See Table 1 for the full explanation of the treatment codes.

Estimates of the shape parameter for each treatment were plotted and differences between treatments were tested using a α^2^ likelihood ratio test (Table 5). There were no detectable effects of interspecific competition on the movement patterns of the urchins (Figure S4 and Table 5). In contrast, there were significantly smaller shape parameters for the distance distributions of abalone after adding urchins and extra abalone (Table 5). This suggests that both intra- and interspecific competition resulted in an increase in the movement of abalone.

### Predators

The number of predators associated with the experimental plots was consistently low throughout the experiment. There were no detectable differences in the total predator densities assessed by divers between treatments in the interspecific competition experiment (*F*_2,28_ = 0.38, p>0.05) or in the intra- and interspecific competition experiment (*F*_2,24_ = 0.87, p>0.05). Similarly, there were no detectable differences among treatments in the densities of rock lobsters, *Jasus edwardsii* or total fishes in the interspecific competition experiment (F_2,28_ = 0.82, p>0.05, F_2,28_ = 0.08, p>0.05, respectively) or the intra- and interspecific competition experiment (F_2,24_ = 459 2.9, p>0.05, F_2,24_ = 0.9, p>0.05, respectively).

## Discussion

The range expansion of highly invasive species to new areas can have major negative impacts on the abundances of native species [7], [15], [18], [19]. We examined the effects of interactions between the range-expanding long spined sea urchin (*Centrostephanus rodgersii*) and the commercially fished black-lipped abalone (*Haliotis rubra*) on their behaviour, movement and local abundances. We extend on previous research on interactions between abalone and sea urchins in experimental enclosures by demonstrating that this urchin has a negative impact on populations of abalone prior to any destructive grazing of seaweeds by the urchin [18], [19].

### Effects of intra- and interspecific competition on abalone

The addition of urchins and extra abalone to plots resulted in a greater proportion of abalone occupying sheltered positions, an increase in the proportion of mobile abalone, and a decrease in the variation of abalone movement within 1 to 2 weeks of the experimental manipulations. Abalone are largely sedentary, feeding by either moving out of cryptic shelters or remaining on their homescars in the open, where they graze attached or trap unattached algae [25], [28], [29]. Research in aquaculture tanks has demonstrated that both juvenile and adult abalone stocked at high densities spend less time out in the open feeding, resulting in declines in their total wet weight, relative to those in tanks at lower densities [29]. The implications are that an increase in the density of competitors in the wild results in abalone altering their behaviour and movement patterns to gain better access to food and/or shelter resources.

The experimental design allowed separation of the relative effects of intra- and interspecific competition on abalone. Relatively few studies thus far have simultaneously tested the effects of intra- and interspecific competition between range expanding and native species [30], [31], [32]. Estimating their relative strengths is essential to assess whether the range expansion of these urchins may result in the local exclusion of abalone. We demonstrated that the effects of the urchins and extra abalone on abalone varied depending on which response variable was considered. Interestingly, additions of either urchins or extra abalone had a similar effect on abalone movement patterns. In contrast, additions of urchins resulted in a greater proportion of abalone moving into shelter or outside the plot and declines in their resightability relative to the treatment with added abalone.

These results could be explained by differences in the mechanisms of competition. An increase in the density of abalone resulted in slower, less consistent changes in abalone behaviour and movement patterns, which could be explained by exploitative competition for food or preferred habitat [28], [29]. In contrast, additions of urchins to plots resulted in abalone immediately changing their behaviour, moving more rapidly and more often moving either deeper into the reef matrix and/or fleeing the experimental plots (realised as a decline in resightability through time), suggesting interference competition for food and/or preferred habitat [17]. The impacts of urchins on abalone could be linked directly to aggressive behaviour such as biting, ‘bulldozing’ by pushing on the shell, or to the presence of their spine canopy [17]. The most important result is that there was clearly a stronger effect of inter- rather than intraspecific competition on the abalone.

We tested the relative effects of intra- and interspecific competition on abalone but not on urchins. This is because there were no detectable effects of removing abalone on urchin behaviour or movement. If we had tested and found there was a significant impact of extra urchins on urchin behaviour and movement this would be an interesting result in itself, but would not influence our conclusions on the interactions between urchins and abalone.

### Effect of abalone on urchins

While it seems clear that urchins negatively affect the behaviour (i.e. microhabitat use), movement and local abundances of abalone, there was no discernible effect of abalone on the urchin. Assuming that abalone do not negatively affect juvenile urchins, which are largely cryptic and restricted to deeper crevices in the reef [13], we found no evidence to suggest that intensive fishing of black-lipped abalone will directly influence the establishment or activity of the urchins.

Our results are consistent with other research investigating interactions between urchins and abalone in intact algal beds [18], [33]. In New Zealand, additions of urchins (*Evechinus chloroticus*) to open plots realised dramatic reductions in the densities of abalone (*Haliotis iris*), while urchin removal resulted in significant increases in abalone densities [33]. In other experiments in Tasmania, adding urchins (*C. rodgersii*) to enclosures resulted in declines in the total weight, dry weight of stomach contents and survival of abalone (*H. rubra*), but no detectable effects of added abalone on the urchins, within six months of the experimental manipulations [18]. These results confirm there are asymmetrical interactions between urchins and abalone [18], [33]. The urchin has a negative impact on abalone but the abalone has no detectable effect on growth, survival or behaviour of the urchin [18], [33].

### Modelling abalone behaviour and movement patterns

This study provides new insights into abalone behaviour and movement patterns. We demonstrated that abalone behaviour at any one time is strongly dependent on their preceding behaviour (first order transition model) and that abalone tended to forage locally with occasional large (and random) ‘jumps’ to new sites (Levy flight model) [34]. Abalone behaviour and movement patterns were correlated with shell length, with a greater proportion of larger abalone spending more time exposed and moving further each week (data not shown). An extension to this study would be to test whether the impacts of adding urchins and extra abalone on abalone behaviour and movement patterns differ between abalone size classes, and whether our results of effects on movement can be explained by competition for food and/or shelter resources.

### Effects of a range expanding urchin on commercial abalone fisheries

Climate change can alter interspecific interactions between species [35], [36], [37], [38], [39]. Our results suggest that establishment of the long-spined urchin in intact algal beds along the east coast of Tasmania causes abalone to seek shelter in cryptic habitat and flee local sites, resulting in increased movement activity and declines in local abundances and resightability. The effects of the urchins on abalone were consistent between seasons, and not attributable to experimental artifacts such as effects of tagging abalone, or to differences in the density of predators between treatments.

Importantly, these urchin-induced shifts in abalone behaviour and movement will reduce the likelihood of abalone detection by fishers. Reduced growth (and possibly survivorship) of abalone in the presence of urchins [18] will also negatively impact this important fishery, which is potentially explained by abalone spending more time in cryptic habitat without readily available food when the urchin is present. Thus, the continued range expansion of this sea urchin along the east coast of Tasmania [14] will result in declines in the abundances and productivity of abalone populations, with concomitant effects on the fishery, even before there is any destructive grazing of seaweeds by the urchin.

We suggest that the establishment of urchins in intact algal beds in eastern Tasmania will initially have a negative impact on the behaviour and movement of abalone through interference competition for food and/or shelter [17]. As the density of the urchins increases at a site, there is a decline in the growth and survival of abalone through exploitative competition for food [17]. As urchin numbers increase at the site, and their destructive grazing results in the formation of urchin barrens, the effects of competitive interactions between urchins and abalone are intensified resulting in the virtual exclusion of abalone from sites on the east coast of Tasmania [17], [18]. Given the high biodiversity and value of species associated with Tasmanian seaweed beds [9] scientists and managers must focus on limiting the establishment or controlling new populations of this urchin in intact algal beds.

### Studying range expansions

It is predicted that many marine species will alter their range in response to global climate change [1], [4]. However, research into interspecific interactions between range expanding and native species is limited [7], [35], [36], [39]. Our study reveals that range expansion of the long-spined sea urchin into Tasmania, Australia will have major negative impacts on valuable commercially fished native abalone species. Our approach including field-based experimental manipulations and Markov chain and Pareto modelling may be widely applicable to the challenging task of predicting the impacts of other range expanding species on native species. Importantly, our results support those from another six month study on the nature and effects of interactions between urchins and abalone growth in experimental enclosures [17]. Thus, assessing the effects of competition on behaviour can be a good proxy for understanding the effects of competition on growth. This approach is likely to be particularly useful in climate change hotspots such as southeast Australia, which is predicted to receive many new species over the coming decades [9], [11].

## Supporting Information

Figure S1 Frequency plots of the distances (m) moved by (a) abalone and (b) urchins per week. Bar widths are 0.05 m. The continuous line shows the probability function that best describes the distribution of the data.
(TIF)

Figure S2 Effects of interspecific competition on the percentage of tagged abalone resighted through time (days), in the Experiment1 at Magistrates Point, Maria Island. Data are the means (+/−SE) of *n* = 3 replicates. Squares are 0U25A: 1× ambient density *H. rubra* and circles are 18U25A: 1× ambient density *H. rubra* with 1× ambient density *C. rodgersii.* There were significant differences between 0U25A vs. 18U25A from day 7 onwards (see Table 2 results).
(TIF)

Figure S3 Effect of interspecific competition on abalone behavioural transitions in Experiment 1, at Magistrates Point, Maria Island. Data are the probability of *H. rubra* in each behavioural state based on the week before (E = exposed, L = lost, O = outside the plot and S = sheltered) in *n* = 3 replicate plots. (a) 0U25A: 1× ambient density *H. rubra*, (weeks 1 to 3 prior to adding urchins and weeks 7 to 9 after urchins were removed combined), (b) 18U25A: 1× ambient density *C. rodgersii* with 1× ambient density *H. rubra*, (1 day after adding urchins), (c) 18U25A: 1× ambient density *C. rodgersii* with 1× ambient density *H. rubra* (1 week after adding urchins), and (d) 18U25A: 1× ambient density *C. rodgersii* with 1× ambient density *H. rubra* (4 weeks after adding urchins). The numbers of *H. rubra* in a given behavioural state are summed across all times for the control and given separately at each time for the treatment and are represented as “n = .” inside each circle. Arrows of different thickness are used to show the probability of abalone transitioning from each behavioural state. The straight arrows show the probability of abalone transitioning from one behavioural state to another (e.g. E to S) and the curved arrows show the probability of abalone remaining in the same behavioural state (e.g. E to E). These probabilities sum to 1 for a given behaviour state (e.g. E-E, E-S, E-L, E-O).
(TIF)

Figure S4 Effect of interspecific competition on the percentage of sedentary (≤0.4 m) and mobile (>0.5) abalone through time (days) in the Experiment 1, at Magistrates Point, Maria Island. (a) 0U18A: (no urchins, 1× ambient density abalone), (b) 18U18A (1× ambient density of urchins added to 1× ambient density abalone). White bars are homing abalone (net distance moved <0.4 m per week) and black bars are mobile abalone (net distances moved ≥0.4 m per week). There were significant differences between 0U18A vs. 18U18A from day 7 onwards (see Table 3 results).
(TIF)

Table S1 Mean (+/−SE) cover of substratum type, algae and sessile invertebrates, and density (m^−2^) of urchins and gastropods in 3×3 m plots at the Lanterns and Magistrates Point.
(DOCX)
