# Supplementary figures and images for: Effects of a Range-Expanding Sea Urchin on Behaviour of Commercially Fished Abalone
Source: PLoS One. 2013 Sep 20;8(9):e73477. doi: 10.1371/journal.pone.0073477 (PMC3779227; doi:10.1371/journal.pone.0073477)

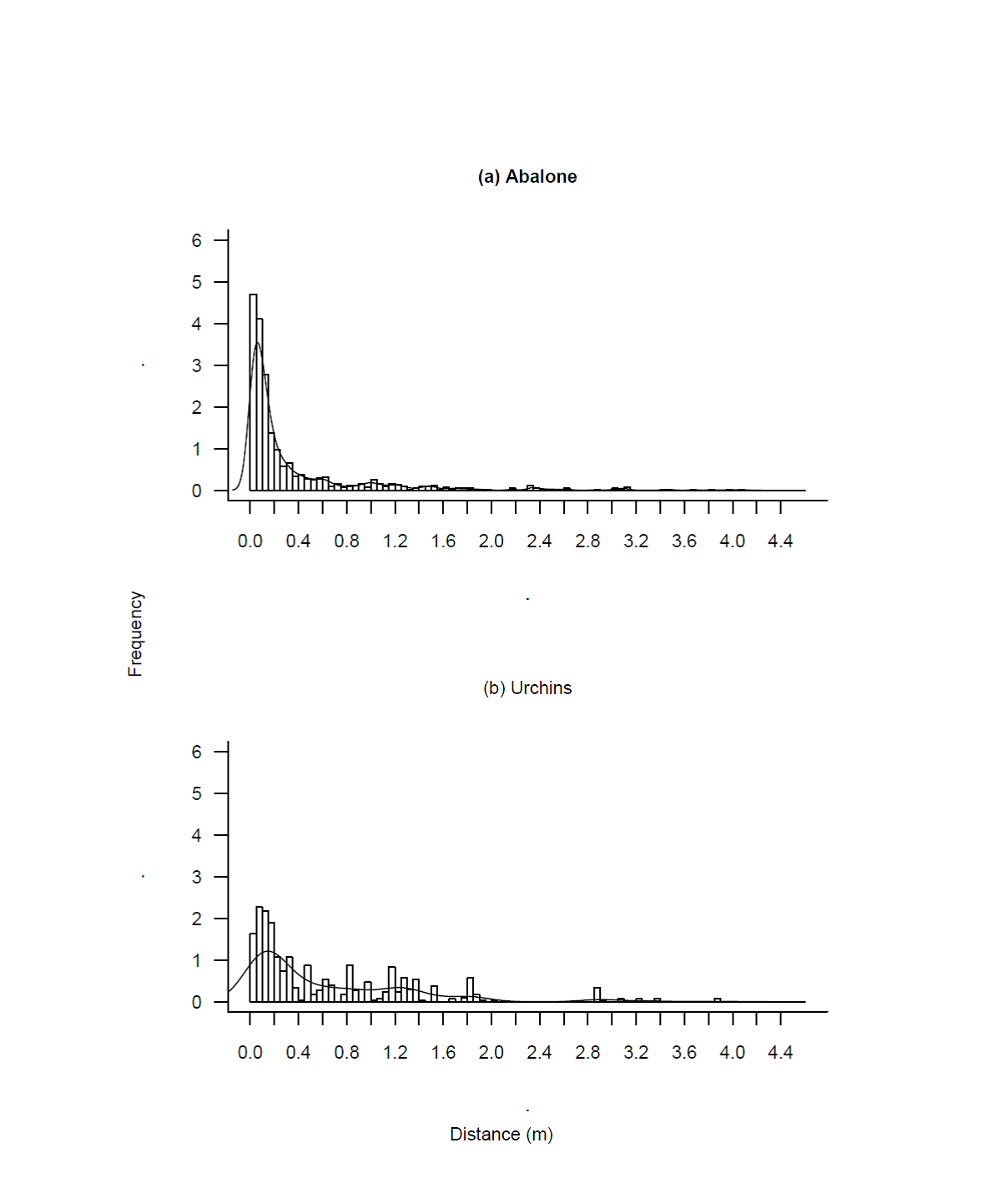

Supplement: Figure S1 — Frequency plots of the distances (m) moved by (a) abalone and (b) urchins per week. Bar widths are 0.05 m. The continuous line shows the probability function that best describes the distribution of the data. (TIF) [file pone.0073477.s001.tif]

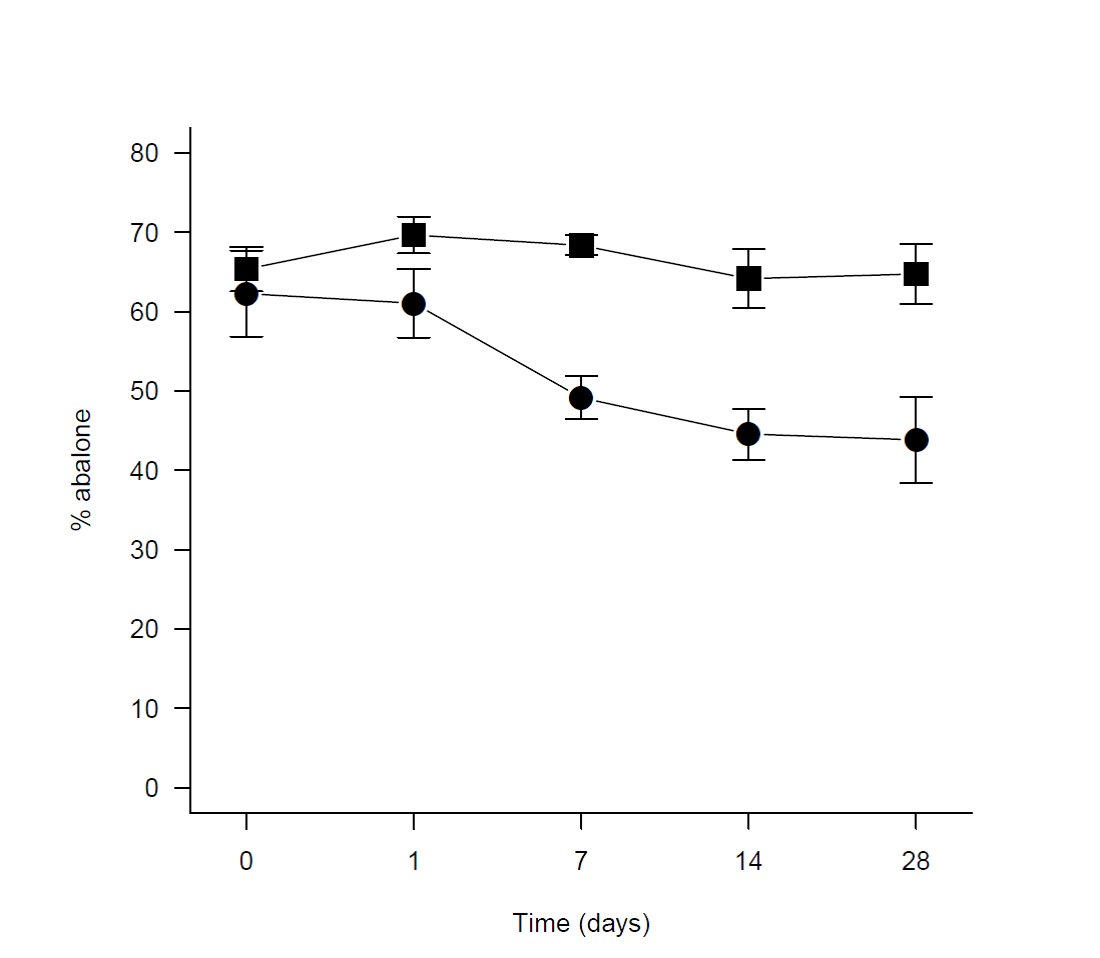

Supplement: Figure S2 — Effects of interspecific competition on the percentage of tagged abalone resighted through time (days), in the Experiment1 at Magistrates Point, Maria Island. Data are the means (+/−SE) of n = 3 replicates. Squares are 0U25A: 1× ambient density H. rubra and circles are 18U25A: 1× ambient density H. rubra with 1× ambient density C. rodgersii. There were significant differences between 0U25A vs. 18U25A from day 7 onwards (see Table 2 results). (TIF) [file pone.0073477.s002.tif]

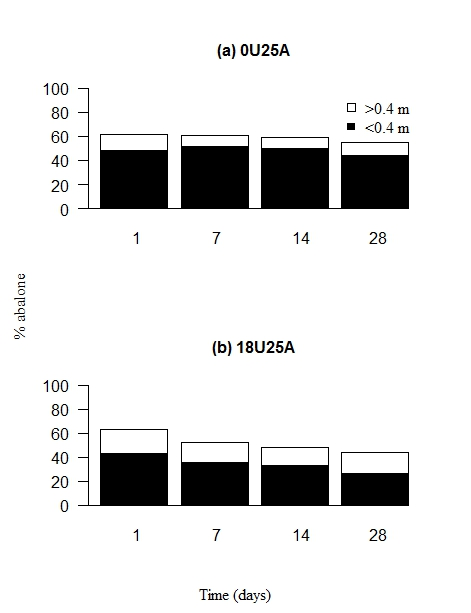

Supplement: Figure S4 — Effect of interspecific competition on the percentage of sedentary (≤0.4 m) and mobile (>0.5) abalone through time (days) in the Experiment 1, at Magistrates Point, Maria Island. (a) 0U18A: (no urchins, 1× ambient density abalone), (b) 18U18A (1× ambient density of urchins added to 1× ambient density abalone). White bars are homing abalone (net distance moved <0.4 m per week) and black bars are mobile abalone (net distances moved ≥0.4 m per week). There were significant differences between 0U18A vs. 18U18A from day 7 onwards (see Table 3 results). (TIF) [file pone.0073477.s004.tif]
