# Supplementary material for: Effects of a Range-Expanding Sea Urchin on Behaviour of Commercially Fished Abalone
Source: PLoS One. 2013 Sep 20;8(9):e73477. doi: 10.1371/journal.pone.0073477 (PMC3779227; doi:10.1371/journal.pone.0073477)
Supplement: Table S1 — Mean (+/−SE) cover of substratum type, algae and sessile invertebrates, and density (m−2) of urchins and gastropods in 3×3 m plots at the Lanterns and Magistrates Point. (DOCX) [file pone.0073477.s005.docx]

| **Site** | **Lanterns** | **Magistrates Point** |
| --- | --- | --- |
| Large boulders >1 to <2.5 m diameter  (% cover) | 67%, +/-2.34% | 17.24%, +/-3% |
| Small boulders >0.2 to <1 m diameter  (% cover) | 5.54%, +/-2.21% | 51.2%, +/-4% |
| Sand (% cover) | 0.54%, +/-0.39% | 9.52%, +/-0.51% |
| Brown algae (% cover) | 48.29%, +/-2.9% | 50%, +/-2.56% |
| Green algae (% cover) | 0% | 24.95%, +/-2.26% |
| Red algae (% cover) | 4.42%, +/-0.86% | 9.12%, +/-0.77% |
| Sessile invertebrates (% cover) | 8.67%, +/-1.86% | 9.11%, +/-0.77% |
| *H. erythrogramma* (m^-2^) | 0.47 m^-2^, +/-0.08 m^-2^ | 0.65 m^-2^, +/-0.22 m^-2^ |
| Gastropods (m^-2^) | 0 | 0.01 m^-2,^ +/-0.001 m^-2^ |
